# Exploring HIV status as a mediator in the relationship of psychological distress with socio-demographic and health related factors in South Africa: findings from the 2012 nationally representative population-based household survey

**DOI:** 10.1186/s12981-022-00498-5

**Published:** 2023-02-06

**Authors:** Nolusindiso Ncitakalo, Lovemore Nyasha Sigwadhi, Musawenkosi Mabaso, John Joska, Leickness Simbayi

**Affiliations:** 1 Medical Education Unit, Walter Sisulu University, Mthatha, South Africa; 2 Division of Epidemiology and Biostatistics, Department of Global Health, Faculty of Medicine and Health Sciences, Stellenbosch University, Cape Town, South Africa; 3 Human and Social Capabilities Research Division, Human Sciences Research Council, Durban, South Africa; 4 HIV Mental Health Research Unit, Department of Psychiatry & Mental Health, University of Cape Town, Cape Town, South Africa; 5 Human Sciences Research Council, Cape Town, South Africa; 6 Department of Psychiatry & Mental Health, University of Cape Town, Cape Town, South Africa

**Keywords:** Psychological distress, HIV status, South Africa, Structural Equation Model

## Abstract

**Background:**

Psychological distress as measured by mental disorders like depression and anxiety is more prevalent in people living with HIV (PLHIV) than in the general population. However, the relationship between mental disorders and HIV is complex and bidirectional. Improved understanding of the relationship between mental disorders and HIV is important for designing interventions for this group. This paper explores the interrelationships of psychological distress with HIV and associated socio-demographic and health-related factors.

**Methods:**

This secondary data analysis used the 2012 South African population-based household survey on HIV collected using a cross-sectional multi-stage stratified cluster sampling design. Generalized structural equation modelling (G-SEM) path analysis was used to explore the direct and indirect relationships of socio-demographic, health and HIV-related factors with psychological distress as measured by Kessler 10 scale using HIV status as a moderator variable.

**Results:**

A total of 20,083 participants were included in the study, 21.7% reported psychological distress, of whom (32.6%) were HIV positive. In the final path model with HIV status as a moderator, psychological distress was significantly more likely among age group 25–49 years (AOR: 1.4 [95% CI 1.3–1.6]), age 50 years and older, (AOR: 1.4 [95% CI 1.2–1.6]), females (AOR: 1.6 [95% CI 1.4–1.8]), high risk drinkers (AOR: 1.9 [1.6–2.2]) hazardous drinkers (AOR: 4.4 [95% CI 3.1–6.3]), ever tested for HIV (AOR: 1.2 [95% CI 1.1–1.3]). Psychological distress was significantly less likely among the married [AOR: 0.8 (0.7–0.9)], other race groups [AOR: 0.5 (0.5–0.6)], those with secondary level education (AOR: 0.9 [95% CI 0.8–0.9]), and tertiary level education (AOR: 0.7 [95% CI 0.6–0.9]), those from rural informal [AOR: 0.8 (0.7–0.9)], and rural formal [AOR: 0.8 (0.7–0.9)] areas and those who rated their health as excellent/good [AOR: 0.4 (0.4–0.5)].

**Conclusion:**

The findings highlight the importance of designing tailored interventions targeted at psychological distress among PLHIV especially the elderly, females, those with no education and / or low education attainment and those residing in informal urban areas.

## Introduction

The comorbidity of HIV and mental disorders has become an increasing major public health challenge and is a substantial burden to society [1]. Common mental disorders are recognized as frequent psychiatric comorbid conditions among PLHIV [2]. Depression is found to be more common in people living with HIV compared with prevalence estimates in the general population [3], directly impacting their quality of life and impeding their enrolment and retention in treatment [4]. Research studies have shown that the causes of mental disorders are multi factorial and include among others biological, social, and economic factors [5].

There is considerable evidence that common mental disorders are distributed according to economic gradient across society and that the poor and disadvantaged suffer disproportionately from common mental disorders and their adverse consequences [5]. Moreover, Knifton and Inglis argue that the mental health of individuals is shaped by the social, environmental and economic conditions in which they are born, grow, work and age [6]. In addition, there is consistent evidence that experience of socioeconomic disadvantage, including unemployment, low income, poverty, debt and poor housing, is associated with poorer mental health [7, 8]. Evidence show that socio-economic conditions and lifestyle factors have a direct influence on the prevalence and severity of mental disorders in both men and women especially among those living with HIV [9].

HIV infection and mental illness are linked in many ways, for example, acquiring HIV can be a serious psychological trauma and can predispose a person to different mental disorders [9]. PLHIV are additionally affected due to lack of social support, poor self-esteem, stigma, and discrimination [10, 11]. This, in turn, predisposes them to psychological problems like depression and anxiety at greater rates than the general population [12, 13]. In addition, HIV-related stigma has been recognised as a fundamental cause of health inequalities [14]. HIV-related stigma has been observed as a contributing factor to mental health and substance use problems among people living with HIV [15]. In addition, HIV testing, and awareness of HIV positive status affects mental capacity to cope especially because of social stigma associated with living with HIV [16]. Since HIV stigma and discrimination affect the emotional well-being and mental health, these feelings can keep people from getting tested and treated for HIV [16, 17].

Reducing the burden of comorbid mental disorders is key to achieving the UNAIDS care cascade goals of 95–95–95 [18]. Mental disorders have been recognized as a risk factor for HIV transmission, through their effects on various aspects of sexual and health seeking behaviour [19–21]. Evidence shows that mental disorders can increase risk of HIV acquisition through both direct and indirect pathways [22]. Regarding direct pathways, several studies have shown that sexually active people with mental disorders have higher risk sexual behavior, including inconsistent condom use, having multiple sexual partners, trading sex, and drinking alcohol before sex [23–25]. Indirect pathways include multiple co-occurring conditions such as mental disorders, substance use disorder, and posttraumatic stress emanating from physical, sexual and / or emotional abuse [20, 21]. Both mental disorders and substance use disorders are known predictors of poor HIV disease management including suboptimal adherence to antiretroviral therapy (ART) and faster disease progression [20, 21]. Other studies suggest that the relationship between mental disorders and HIV/AIDS is complex and bidirectional [26].

Many factors contribute to the high comorbidity of HIV and mental health conditions. However, the underlying factors remain poorly understood. Elsewhere, studies have used structural equation model (SEM) to understand this complex relationship by investigating a conceptual model of the pathways linking wellbeing including mental health, social support, self-rated health and HIV-related stigma [26]. SEM has been utilized to develop psychological model to predict antiretroviral therapy medication adherence behavior [27]. Others have used SEM to investigate factors associated with HIV risk behaviors and mental health and examine the role of intersecting stigmas [28]. However, in sub-Saharan African countries including South Africa there is paucity of large population-based studies of complex interactions between psychological distress, HIV status and predisposing factors.

This paper therefore explores the relationship of psychological distress with HIV status and associated socio-demographic, health related factors in South Africa using the 2012 nationally representative household-based population survey on HIV.

## Methodology

### Data source

This secondary data analysis used the 2012 South African population-based household survey on HIV [29]. The data was collected using a multi-stage stratified cluster sampling design. A total of 1000 census enumeration areas (EAs) from the 2001 population census in South Africa were randomly selected using probability proportional to size and stratified by province, locality type and race in urban areas from a database of 86,000 EAs. In each sampled EA a total of 15 visiting points (VPs) or households were used as secondary sampling units. Persons of all ages living in South African households and hostels were eligible to participate and formed the ultimate sampling unit.

Four questionnaires including a household questionnaire and three age-appropriate individual questionnaires were used for data collection. These questionnaires were translated into main languages spoken in the nine provinces across the country and administered by trained fieldworkers. Fieldworkers were trained on community entry, obtaining informed consent/assent, conducting interviews, maintaining confidentiality, ethical procedures, collection of dried blood spot (DBS) specimen for laboratory testing and quality control procedures. The questionnaires solicited among others information about socio-demographic characteristics, sexual behaviors, knowledge, beliefs, and practices related to HIV including HIV related stigma and discrimination against PLHIV.

In addition, blood specimens were collected from consenting individuals for HIV testing using DBS. Blood samples were tested for HIV using an enzyme immunoassay (EIA) (Vironostika HIV Uni-Form II plus O, Biomeriux, Boxtel, The Netherlands), and samples which tested positive were retested using a second EIA (Advia Centaur XP, Siemens Medical Solutions Diagnostics, Tarrytown, New York, USA). Any samples with discordant results on the first two EIAs were tested with a third EIA (Roche Elecys 2010 HIV Combi, Roche Diagnostics, Mannheim, Germany). The current study is based on a sub-sample of youth and adult individuals 15 years and older who responded to the questions on psychological distress.

### Ethical consideration

Ethical approval for the study was obtained from the Research Ethics Committee of the Human Sciences Research Council, South Africa (REC: 5/17/11/10) as well as by the Associate Director of Science of the National Center for HIV and AIDS, Viral Hepatitis, STD and TB Prevention at the USA’s Centers for Disease Control and Prevention (CDC) in Atlanta, Georgia, USA. All persons who agreed to participate in the survey were required to provide either written or verbal consent for both the interview and specimen collection. Parents and guardians of children under 18 years of age were asked to give informed consent for inclusion of their children in the survey. Children under 18 years were required to confirm their assent by placing a tick or cross in a demarcated box in addition to providing written consent by means of a signature (where possible).

## Measures

### Endogenous variables

Psychological distress was the observed endogenous variable based on the respondent’s experience of depressive and anxiety disorders measured using The Kessler Psychological Distress Scale (K10) [30]. This scale has been validated among low- and middle-income countries including South Africa [31, 32]. This the scale consists of the following 10 items that describe how they felt during the previous 30 days: How often did you feel: Tired out for no good reason? So nervous that nothing could calm you down? Hopeless; Restless or fidgety: So restless that you could not sit still; Depressed? That everything was an effort? So sad that nothing could cheer you up? Worthless?’ Responses to these items were recorded using a 5-point Likert scale (1 = never, 2 = rarely, 3 = some of the time, 4 = most of the time, 5 = all of the time). The raw scores were summed, and a total score grouped into four categories that indicated that respondents were likely to be well (score below 20), experiencing mild (score 20–24), moderate (score 25–29) or severe (score 30 and above) psychological distress [33]. The scores were then dichotomized into a binary outcome those who scored < 19 (absence of psychological distress = 0) and those who scored ≥ 20 (presence of psychological distress = 1). The internal reliability coefficient for the K-10 in this study was Cronbach alpha = 0.90.

### Exogenous variables

The selected exogenous variables included a set of demographic variables such as age (15–24, 25–34, 35–49, 50 years and older), sex (male and female), race (Black African and other races), educational level (primary/no education, secondary, tertiary), employment status (unemployed and employed), locality type (urban formal, urban informal, rural informal/ tribal areas, rural formal/farm areas) [34] and asset based socio-economic status constructed using multiple correspondence analyses (MCA) based on questions on availability/ownership of broad range of household assets ownership and access to utilities. MCA calculated a composite indicator score computed by adding up all weighted responses [35]. The predicted score for each household was used to compute five quintiles (1st lowest, 2nd lower, 3rd middle, 4th higher and 5th highest) representing a continuum of household SES from the poorest to the least poor. These were then dichotomised into low SES (lowest 3 quintiles) and high SES (highest 2 quintiles).

This also included HIV-related variables such as self-perceived risk of contracting HIV infection (no and yes), HIV knowledge and myth rejection (no and yes), ever tested for HIV (no and yes), correct HIV knowledge and myth rejection based on responses from the following questions: (Can AIDS be cured? Can a person reduce the risk of HIV by having fewer sexual partners? Can a healthy-looking person have HIV? Can a person get HIV by sharing food with someone who is infected? Can a person reduce the risk of getting HIV by using a condom every time he/she has sex? (no and yes), awareness of HIV status based on the question “Have you been told/informed of the result of your most recent test? (no and yes), external HIV-related stigma (yes and no), self-rated health (fair/poor and good/excellent), based on the Alcohol Use Disorder Identification Test (AUDIT) score (0 = abstainers; 1–7 = low-risk drinkers; 8–19 = high-risk drinkers; 20+  = hazardous drinking) [36].

### Mediator variable

HIV status was included as a mediator in the relationship between the endogenous and exogenous variables. It is hypothesized that HIV status mediates the effects of demographic, health and HIV-related variables on psychological distress.

### Conceptual model and analysis

**Fig. 1:**
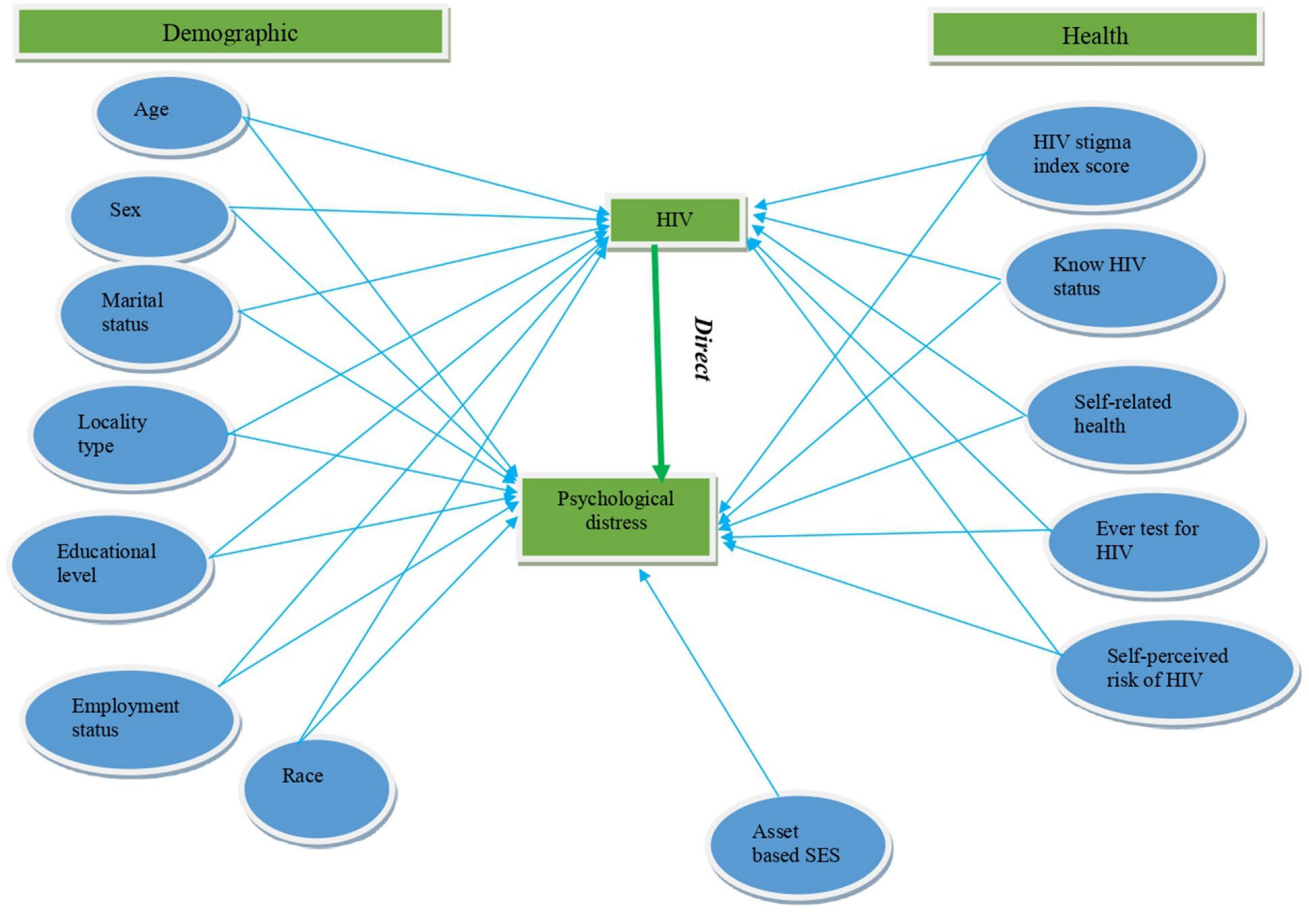
Conceptual model of the relationship between psychological distress, mediator variable HIV status, socio-demographic, health and HIV-related variables

Generalized structural equation modelling (G-SEM)-path analysis was used to explore the direct and indirect relationships of key variables with psychological distress using HIV status as a mediator variable (see Fig. 1). The conceptual model follows the Fundamental Causes Theory which suggests that individuals’ health condition is influenced by contextual factors [31] such as demographics (age, gender, race, locality), socio-economic status (educational level, employment), social contexts (social support), and persistent health disparities (self-rated health, HIV related stigma). This model also includes health and HIV-related factors such alcohol use AUDIT score, self-rated health, HIV testing history (ever had an HIV test), awareness of HIV status, self-perceived risk of HIV, and experiences of externalised HIV-related stigma.

G-SEM was used to measure linear and non-linear causal relationships among selected variables, while simultaneously accounting for measurement error. G-SEM is a combination of three statistical techniques: multiple regression, path analysis, and factor analysis. Its purpose was to determine the extent to which a proposed theoretical model, expressed by a set of relations among different constructs, is supported by the collected data. Parameters from a G-SEM model are constants and indicate the nature and size of the relationship between two variables being assessed. Mediation analysis for each variable was performed and a final path analysis including the goodness of fit was conducted. Goodness-of-fit chi square test, root mean square error of approximation (RMSEA), Tucker–Lewis’s index (TLI), and comparative fit index (CFI) were used to assess the model fit. All variables with p < 0.05 were considered statistically significant and statistical analyses were performed using Stata (V.16, Stata Corp, College Station, Texas, USA) statistical software.

## Results

### Characteristics of the study sample

Table 1 shows that most participants were aged 25 to 49 years, female, not married, Black African, had secondary level education, unemployed, from low SES households, resided in urban areas, abstained from alcohol, rated their health as good/excellent, reported ever testing for HIV, not aware of their HIV status, perceived themselves as being at risk of HIV, and about half reported experiences of externalised HIV related stigma.

**Table 1:** Socio-demographic, health and HIV-related characteristics of the study sample (n = 20,083)

Variables	Study sample	
	Total	%
**Age group (years)**		
15 to 24	5716	28.5
25 to 49	8573	42.7
50+	5789	28.8
**Sex**		
Male	8503	42.3
Female	11,580	57.7
**Race group**		
African	12,097	60.3
Other	7970	39.7
**Marital status**		
Not married	13,235	66.8
Married	6568	33.2
**Educational level**		
No education/Primary	3663	21.4
Secondary	12,104	70.7
Tertiary	1348	7.9
**Employment status**		
No	11,455	62.6
Yes	6849	37.4
**Asset based SES**		
Low SES	10,856	54.7
High SES	9003	45.3
**Locality type**		
Urban formal	11,080	55.2
Urban informal	2159	10.8
Rural informal	4696	23.4
Rural formal	2148	10.7
**Alcohol use AUDIT score**		
Abstainers	11,466	64.0
Low risk drinkers (1–7)	4759	26.6
High risk drinkers (8–19)	1498	8.4
Hazardous drinkers (20+)	198	1.1
**Self-rated health**		
Fair/poor	4149	20.7
Good/excellent	15,872	79.3
**Ever had HIV test**		
No	7372	36.8
Yes	12,637	63.2
**Awareness of HIV status**		
No	12,045	60.8
Yes	7769	39.2
**Self-perceived Risk of HIV infection**		
No	3995	20.1
Yes	15,896	79.9
**Externalised HIV related stigma**		
No	9606	48.2
Yes	10,312	51.8

*SES* Socio economic status, *AUDIT* Alcohol risk score based on a questionnaire for Alcohol Use Disorder Identification Test and scores used for categorisation within parentheses. Subtotals do not total (n) due to non-response and/or missing data

### Psychological distress and sample characteristics

Table 2 shows that out of the 20 083 participants (22.7%) had psychological distress, of whom (32.6%) were HIV positive. Psychological distress was significantly higher among those aged 50+ years (26%), among females (26.3%), those not married (23.8%), Black African (26.8%), those with no education (27.4%), those from informal urban areas (28.6%), those who abstained from alcohol (66.6%), those who reported bad/poor self-rated health (39.3%), those who ever tested for HIV (23.9%), those aware of their HIV status (24.3%) and those who perceived themselves as being not at risk of HIV (28.9%).

**Table 2:** Psychological distress by socio-demographic, health and HIV-related characteristics, South Africa 2012

Sample characteristic	Total (n = 20,083)	Psychological distress		p-value
		No (n = 15,527)	Yes (n = 4556)	
**Age group (years)**				< 0.001
15 to 24	5716	4696 (82.2%)	1020 (17.8%)	
25 to 49	8573	6544 (76.3%)	2029 (23.7%)	
50+	5789	484 (74.0%)	1505 (26.0%)	
**Sex**				< 0.001
Male	8503	692 (82.2%)	1511 (17.8%)	
Female	11,580	8535 (73.7%)	3045 (26.3%)	
**Race group**				< 0.001
African	12,097	8853 (73.2%)	3244 (26.8%)	
Other	7970	6661 (83.6%)	1309 (16.4%)	
**Marital status**				< 0.001
Not Married	13,235	10,086 (76.2%)	3149 (23.8%)	
Married	6568	5236 (79.7%)	1332 (20.3%)	
**Educational level**				< 0.001
No education/Primary	3663	2659 (72.6%)	1004 (27.4%)	
Secondary	12,104	9583 (79.2%)	2521 (20.8%)	
Tertiary	1348	1138 (84.4%)	210 (15.6%)	
**Employment status**				< 0.001
No	11,455	8565 (74.8%)	2890 (25.2%)	
Yes	6849	5623 (82.1%)	1226 (17.9%)	
**Asset-based SES**				< 0.001
Low SES	10,856	8059 (74.2%)	2797 (25.8%)	
High SES	9003	7295 (81.0%)	1708 (19.0%)	
**Locality type**				< 0.001
**Alcohol use AUDIT score**				< 0.001
Abstainers	11,466	8739 (76.2%)	2727 (23.8%)	
Low risk drinkers (1–7)	4759	3947 (82.9%)	812 (17.1%)	
High risk drinkers (8–19)	1498	1047 (69.9%)	451 (30.1%)	
Hazardous drinkers (20+)	198	91 (46.0%)	107 (54.0%)	
**Self-rated health**				< 0.001
Fair/poor	4149	2518 (60.7%)	1631 (39.3%)	
Good/excellent	15,872	12,966 (81.7%)	2906 (18.3%)	
**Ever had HIV test**				< 0.001
No	7372	5857 (79.4%)	1515 (20.6%)	
Yes	12,637	9614 (76.1%)	3023 (23.9%)	
**HIV status**				< 0.001
Negative	17,546	13,816 (78.7%)	3730 (21.3%)	
Positive	2537	1711 (67.4%)	826 (32.6%)	
Urban formal	11,080	8734 (78.8%)	2346 (21.2%)	
Urban informal	2159	1541 (71.4%)	618 (28.6%)	
Rural informal	4696	3512 (74.8%)	1184 (25.2%)	
Rural formal	2148	1740 (81.0%)	408 (19.0%)	
**HIV stigma index score**				0.650
No	9606	7413 (77.2%)	2193 (22.8%)	
Yes	10,312	7986 (77.4%)	2326 (22.6%)	
**Self-perceived** **Risk of HIV infection**				< 0.001
No	3995	2842 (71.1%)	1153 (28.9%)	
Yes	15,896	12,555 (79.0%)	3341 (21.0%)	
**Awareness of HIV status**				< 0.001
No	12,045	9431 (78.3%)	2614 (21.7%)	
Yes	7769	5884 (75.7%)	1885 (24.3%)	

*SES* Socio economic status, *AUDIT* Alcohol risk score based on a questionnaire for Alcohol Use Disorder Identification Test and scores used for categorisation within parentheses. Subtotals do not total (n) due to non-response and/or missing data

### HIV status, psychological distress and sample characteristics

Table 3 shows that 4556 participants were psychologically distressed and 18.1% of them were HIV positive. The proportion of HIV positive and psychological distressed patients was higher in females than males (20.2% vs 14%). Lack of education showed that those without education/primary had highest proportion of HIV positive (19.4%), followed by those with secondary education with a decline of only 0.5%. However, participants with tertiary education were twice less likely to be HIV positive (9%). Married participants had lower proportion than the not married group (21.6% vs 9.2%). High proportion of HIV positive patients was among those experiencing HIV stigma (21.6% vs 14.5%) and higher among those who had never had an HIV test (23.1% vs 8.5%).

**Table 3:** HIV status among participants with psychological distress by socio-demographic, health and HIV-related characteristics, South Africa 2012

Sample characteristic	Total (n = 4556)	HIV status		p-value
		Negative (n = 3730)	Positive ((n = 826)	
**Age group (years)**				< 0.001
15 to 24	1020 (22.4)	919 (90.1)	101 (9.9)	
25 to 49	2029 (44.6)	1430 (70.5)	599 (29.5)	
50+	1505 (33.0)	1379 (91.6)	126 (8.4)	
**Sex**				< 0.001
Male	1511 (33.2)	1299 (86.0)	212 (14.0)	
Female	3045 (66.8)	2431 (79.8)	614 (20.2)	
**Race group**				< 0.001
African	3244 (71.2)	2482 (76.5)	762 (23.5)	
Other	1309 (28.8)	1245 (95.1)	64 (4.9)	
**Marital status**				< 0.001
Not Married	3149 (70.3)	2469 (78.4)	680 (21.6)	
Married	1332 (29.7)	1209 (90.8)	123 (9.2)	
**Educational level**				0.001
No education/Primary	1004 (26.9)	809 (80.6)	195 (19.4)	
Secondary	2521 (67.5)	2044 (81.1)	477 (18.9)	
Tertiary	210 (5.6)	191 (91.0)	19 (9.0)	
**Employment status**				< 0.001
No	2890 (70.2)	2353 (81.4)	537 (18.6)	
Yes	1226 (29.8)	976 (79.6)	250 (20.4)	
**Asset based SES**				< 0.001
Low SES	2797 (62.1)	2145 (76.7)	652 (23.3)	
High SES	1708 (37.9)	1548 (90.6)	160 (9.4)	
**Locality type**				< 0.001
Urban formal	2346 (51.5)	2065 (88.0)	281 (12.0)	
Urban informal	618 (13.6)	454 (73.5)	164 (26.5)	
Rural informal	1184 (26.0)	899 (75.9)	285 (24.1)	
Rural formal	408 (9.0)	312 (76.5)	96 (23.5)	
**Alcohol use AUDIT score**				0.001
Abstainers	2727 (66.6)	2182 (80.0)	545 (20.0)	
Low risk drinkers (1–7)	812 (19.8)	693 (85.3)	119 (14.7)	
High risk drinkers (8–19)	451 (11.0)	383 (84.9)	68 (15.1)	
Hazardous drinkers 20 +)	107 (2.6)	87 (81.3)	20 (18.7)	
**Self- rated health**				< 0.001
Fair/ Poor	1631 (35.9)	1273 (78.1)	358 (21.9)	
Good/ Excellent	2906 (64.1)	2438 (83.9)	468 (16.1)	
**Ever had HIV test**				< 0.001
No	3023 (66.6)	2326 (76.9)	697 (23.1)	
Yes	1515 (33.4)	1386 (91.5)	129 (8.5)	
**Awareness of HIV status**				< 0.001
No	2614 (58.1)	2224 (85.1)	390 (14.9)	
Yes	1885 (41.9)	1463 (77.6)	422 (22.4)	
**Self-percieved risk of HIV infection**				< 0.001
No	1153 (25.7)	695 (60.3)	458 (39.7)	
Yes	3341 (74.3)	3001 (89.8)	340 (10.2)	
**Externalsed HIV related stigma**				< 0.001
No	2193 (48.5)	1874 (85.5)	319 (14.5)	
Yes	2326 (51.5)	1824 (78.4)	502 (21.6)	

*SES* Socio economic status, *AUDIT* Alcohol risk score based on a questionnaire for Alcohol Use Disorder Identification Test and scores used for categorisation within parentheses. Subtotals do not total (n) due to non-response and/or missing data

### Factors associated with psychological distress

Table 4 shows how the exogenous variables influence psychological distress (Step 1 in establishing mediation). Most independent variables significantly influenced psychological distress. Those aged 25 to 45 years, 50 years and older, females and those residing in rural formal and rural informal areas were significantly more likely to develop psychological distress compared to urban formal areas. Those who drink alcohol (low risk drinker, high risk drinker and hazardous drinkers), and those who ever tested for HIV, were also significantly more likely to develop psychological distress compared to their counterparts. In addition, those married were less likely to develop psychological distress compared to the unmarried. Participants with secondary and tertiary level education were significantly less likely to develop psychological distress compared to those with no education.

**Table 4:** Model of the relationship of psychological distress with socio-demographic, health and HIV-related variables

Psychological distress	OR	95% CI		p-value
**Age group (years)**				
15–24	Ref			
25 to 49	1.49	1.33	1.67	< 0.001
50+	1.42	1.23	1.63	< 0.001
**Sex**				
Male	Ref			
Female	1.63	1.48	1.80	< 0.001
**Race group (years)**				
African	Ref			
Other	0.53	0.47	0.60	< 0.001
**Marital status**	Ref			
Not married				
Married	0.80	0.72	0.89	< 0.001
**Educational level**				
Primary	Ref			
Secondary	0.88	0.79	0.98	0.004
Tertiary	0.70	0.57	0.87	< 0.001
**Employment status**				
No				
Yes	0.78	0.70	0.86	< 0.001
**Asset based SES**				
Low	ref			
High	1.01	0.90	1.14	0.884
**Locality type**				
Urban formal	Ref			
Urban informal	0.97	0.84	1.13	0.686
Rural informal	0.78	0.68	0.89	< 0.001
Rural formal	0.79	0.66	0.92	0.002
**Alcohol use AUDIT score**				
Abstainers	Ref			
Low risk drinkers (1–7)	0.90	0.81	1.01	0.071
High risk drinkers (8–19)	1.88	1.61	2.20	< 0.001
Hazardous drinkers (20+)	4.43	3.12	6.27	< 0.001
**Self-rated health**				
Fair/poor	Ref			
Good/excellent	0.40	0.37	0.45	< 0.001
**Ever test for HIV**				
No				
Yes	1.16	1.04	1.29	0.008
**Awareness of HIV status**				
No	Ref			
Yes	1.02	0.91	1.13	0.786
**Self-perceived risk of HIV**				
No	Ref			
Yes	0.92	0.82	1.02	0.111
**Externalised HIV related stigma**				
No	Ref			
Yes	0.93	0.85	1.02	0.111

*SES* Socio economic status, *AUDIT* Alcohol risk score based on a questionnaire for Alcohol Use Disorder Identification Test and scores used for categorisation within parentheses, OR Odds ratio, *CI* confidence intervals. Subtotals do not total (n) due to non-response and / or missing data

Table 5 shows how the exogenous variables influence the mediator variable HIV status (Step 2 in establishing mediation). Those aged 25 to 45 years, 50 years and older were significantly more likely to be HIV positive compared to 15 to 24 years. Similarly, participants residing in informal and formal rural areas were likely to be HIV positive than formal urban participants. Those who ever tested for HIV were more likely to be HIV positive than those who had never tested. Furthermore, those married, were significantly less likely to develop psychological distress compared to the unmarried group. Those who rated their health as excellent/good were also significantly less likely to develop psychological distress compared to poor self-rated health.

**Table 5:** Model of the relationship of the mediator variable HIV status with socio-demographic, health and HIV related variables

HIV status	OR	95% CI		p-value
**Age groups (years)**				
15–24	Ref			
25 to 49	3.84	3.29	4.47	< 0.001
50+	1.50	1.21	1.87	< 0.001
**Sex**				
Male	Ref			
Female	1.57	1.37	1.80	< 0.001
**Race group**				
African	Ref			
Other	0.19	0.15	0.23	< 0.001
**Marital status**				
Not married	Ref			
Married	0.41	0.36	0.48	< 0.001
**Educational level**				
Primary	Ref			
Secondary	0.94	0.82	1.09	0.416
Tertiary	0.44	0.32	0.59	< 0.001
**Employment statu**s				
No	Ref			
Yes	0.94	0.82	1.09	0.416
**Asset based SES**				
Low				
High				
**Locality type**				
Urban formal	Ref			
Urban informal	1.48	1.25	1.75	< 0.001
Rural informal	1.22	1.05	1.42	0.009
Rural formal	1.60	1.31	1.94	< 0.001
**Alcohol use AUDIT score**				
Abstainers	Ref			
Low risk drinkers (1–7)	0.93	0.81	1.08	0.358
High risk drinkers (8–19)	1.00	0.80	1.25	0.967
Hazardous drinkers (20+)	1.50	0.92	2.44	0.102
**Self-related health**				
Fair/poor	Ref			
Good/excellent	0.68	0.59	0.78	< 0.001
**Ever test for HIV**				
No	Ref			
Yes	2.15	1.83	2.54	< 0.001
**Awareness of HIV status**				
No	Ref			
Yes	0.75	0.66	0.86	< 0.001
**Self-perceived risk of HIV**				
No	Ref			
Yes	0.41	0.36	0.46	< 0.001
**Externalised HIV related stigma**				
No	Ref			
Yes	1.24	1.10	1.39	< 0.001

*SES* Socio economic status, *AUDIT* Alcohol risk score based on a questionnaire for Alcohol Use Disorder Identification Test and scores used for categorisation within parentheses, OR Odds ratio, *CI* confidence intervals. Subtotals do not total (n) due to non-response and/or missing data

**Fig. 2:**
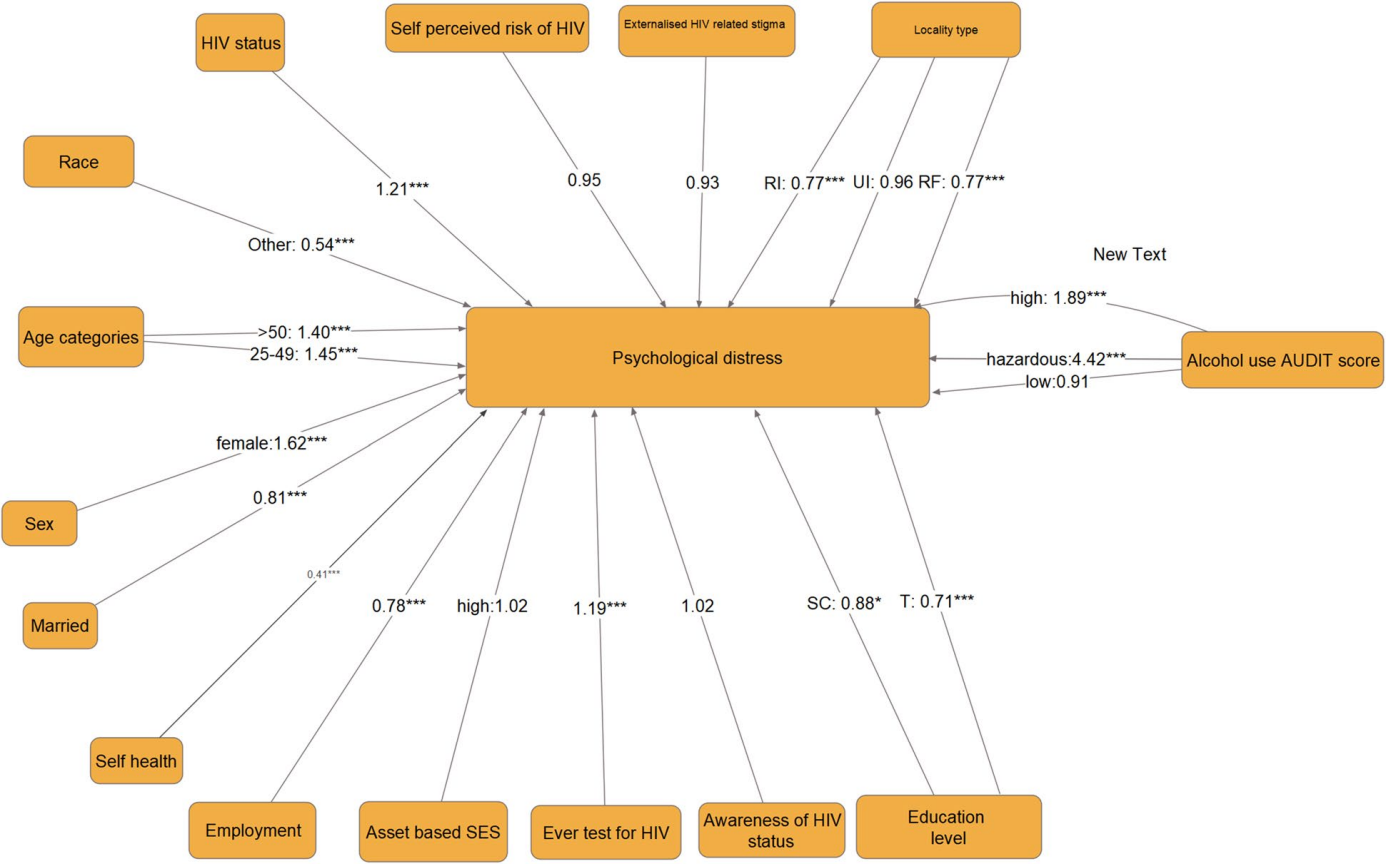
Final path model of the relationship between psychological distress, mediator variable, socio-demographic, health and HIV related variables

Figure 2 shows how the mediator variable influences psychological distress (Step 3 in establishing mediation). The final model shows that HIV significantly influenced psychological distress levels in the third equation. A strong association between most of the exogenous variables and psychological distress was observed, thus second condition satisfied. HIV status was a strong predictor of psychological distress therefore third condition satisfied.

In the final model (Table 6), those aged 25 to 49 years and 50 years and older were more likely to develop psychological distress compared to those aged 15–24 years. Females were more likely to develop psychological distress compared to males. Those residing in rural areas were significantly more likely to develop psychological distress compared to those residing in urban formal areas. Those who ever tested for HIV were more likely to develop psychological distress compared to those who had never tested. Furthermore, high risk and hazardous alcohol drinkers were more likely to develop psychological distress. Psychological distress was significantly less likely among married participants compared to unmarried participant, those with secondary and tertiary educational level compared to no education, the employed compared to the unemployed and those who rated their health as good/excellent comapred to those who rated their health as fair/poor.

**Table 6:** Model of the relationship of psychological distress with socio-demographic, health, HIV-related variables and HIV status as a mediator

Psychological distress	OR	95% CI		p-value
HIV status: Positive	1.213	1.068	1.377	0.003
**Age group (years)**				
15–24	Ref			
25 to 49	1.445	1.285	1.626	< 0.001
50+	1.399	1.213	1.612	< 0.001
**Sex:** Female	1.624	1.470	1.794	< 0.001
**Race:** African				
Other	0.539	0.478	0.608	< 0.001
**Marital status:** Married	0.813	0.730	0.905	< 0.001
**Educational level**				
Primary	Ref			
Secondary	0.880	0.786	0.984	0.025
Tertiary	0.712	0.578	0.877	0.001
**Employment status:** Employed	0.778	0.702	0.861	< 0.001
**Asset based SES:** High	1.015	0.901	1.143	0.805
**Locality type**				
Urban formal	Ref			
Urban informal	0.960	0.827	1.115	0.596
Rural informal	0.774	0.679	0.883	< 0.001
Rural formal	0.773	0.656	0.911	0.002
**Alcohol use AUDIT score**				
Abstainers	Ref			
Low risk drinkers (1–7)	0.906	0.811	1.012	0.079
High risk drinkers (8–19)	1.885	1.612	2.204	< 0.001
Hazardous drinkers (20 +)	4.418	3.116	6.265	< 0.001
**Self-related health: Good/Excellent**	0.409	0.369	0.455	< 0.001
**Ever test for HIV: Yes**	1.187	1.052	1.340	0.005
**Know HIV results: **Yes	1.024	0.917	1.142	0.676
**Self-perceived risk of HIV: **Yes	0.945	0.847	1.053	0.302
**Externalsed HIV related stigma:** Yes	0.926	0.848	1.011	0.087

*SES* Socio-economic status, *AUDIT* Alcohol risk score based on a questionnaire for Alcohol Use Disorder Identification Test and scores used for categorisation within parentheses, OR Odds ratio, *CI* confidence intervals. Subtotals do not total (n) due to non-response and/or missing data

Table 7 shows that the introduction of HIV status into the model did not weaken the effect of any independent variables. Slight changes on alcohol use AUDIT score among the hazardous drinkers reduced by almost 2%, those aged 50+ were reduced by 2%, sex by 0.05% reduction. A one percent reduction on rural informal locality type was observed. However, the relationship between educational levels, race, self-perceived risk, knowledge of HIV results, marital status, HIV stigma index score, asset-based SES, self-related health, and ever test for HIV did not differ after the introduction of HIV status. This supported the last condition by Baron and Kenny confirming that HIV status mediates the effect of independent variables towards psychological distress. The likelihood ratio test shows that the p-value is less than 0.05 suggesting that model 2 is better than the previous model, and that our model is a good fit. RMSEA assessed the population error, and it was very small close to zero suggesting a good fit of our model. The same idea was supported by the p-close of 1. TLI value greater than 0.95 provide evidence of the acceptance of model fit.

**Table 7:** The performance of the three fit statistics (Likelihood ratio, RMSEA, TLI, CFI.)

Fit statistic	Value	Description
*Likelihood ratio*		
chi2_ms (2)	3161.428	Model 1 vs. Model 2
p > chi2	< 0.001	
chi2_bs (11)	3049.923	Baseline vs. saturated
p > chi2	< 0.001	
*Population error*		
RMSEA	0.000	Root mean squared error of approximation
90 CI, lower bound	< 0.001	
Upper bound	0.000	
p-close	1.000	Probability RMSEA < = 0.05
*Baseline comparison*		
CFI	1.000	Comparative fit index
TLI	1.000	Tucker-Lewis index

## Discussion

In this study, we examined the interrelationships between psychological distress, HIV status and associated factors among youth and adults 15 years and older using data from a nationally representative cross-sectional survey. This is the first study that explored the complex and bidirectional relationship between psychological distress and HIV status and associated predisposing factors to both conditions. The prevalence of psychological distress among the study population was 21.7%, and of these 12.6% were HIV positive.

The final model with HIV status as a mediator variable showed that other than HIV infection, psychological distress was significantly associated with older age group than youth (15–24 years and female. There is lack of consistent results about how age affects depression and anxiety [37]. Contrary to current findings, other studies found lower level of distress in older age groups [38]. In South Africa, HIV is a major problem among the youth especially among females [34]. Other studies have also found a higher prevalence of psychological distress among women living with HIV compared to men. It is likely therefore that high levels of HIV infection among the youth and females predispose these population groups to psychological distress. These observations emphasize the need for differentiated care and targeted interventions to support these vulnerable groups.

Furthermore, the model showed that heavy alcohol intake was associated with psychological distress. Other studies have also found that harmful lifestyle factors such as excessive drinking in PLHIV increased risk for anxiety and depressive symptoms [36]. This suggests that interventions should mitigate the effects of adverse lifestyle factors such as alcohol abuse in preventing psychological distress especially among PLHIV. The current findings therefore highlight the importance of screening for alcohol abuse in this group.

In agreement with current findings, other studies found that HIV-infected individuals with psychological distress were more likely to have had an HIV test, partly due to worry arising from the knowledge of potentially being infected [39]. Evidence shows that the impact of being diagnosed with HIV infection, associated stigma, social isolation, and discrimination may all lead to depressive disorders among PLHIV. However, others argue that symptoms of depression and distress are common among persons seeking HIV testing and are therefore not a consequence of an HIV-positive test result [40]. Nevertheless, the findings of this study support proposals for greater integration of mental health services with HIV testing services especially in populations suffering from high levels of psychological distress.

The finding that marriage is protective of psychological distress is consistent with other studies indicating a benefit of marriage for mental health partly due to family/social support since marriage protects against feelings of loneliness [41]. Other studies also found that those in marriage suffer less psychological distress and have higher levels of emotional and psychological well-being than those who are single, divorced, or cohabiting [42]. The observed positive influence of marriage on psychological distress highlights the importance of improving marital quality to promote mental health.

The observed differences in psychological distress between Black Africans and other race groups in the context of HIV can be attributed to the racial disparities rooted in structural and contextual inequalities that sustain the HIV epidemic among Black Africans [43]. Other studies also observed that socio-economic status help explain differences between Black Africans and other race groups [44]. This suggest that addressing social and resource inequality such as access to basic services, education, and employment will in a way address social stressors and mitigate psychological distress especially among PLHIV.

The finding of protective effects of socio-economic status indicators such as education attainment and employment against psychological distress is partly because educational achievement has a positive effect on outlook in life and increase self-efficacy, which in turn helps people cope with life’s problems and stresses [45]. On the other hand, employment promotes positive emotions due to social security since jobs provide resources that can mitigate stress, support healthy lifestyles and thereby promote mental health [46]. Therefore, policies promoting access to education and reducing unemployment may be important for mitigating the impact of psychological distress especially among PLHIV.

The findings also suggest that the relationship between HIV and psychological distress differs across urban and rural settings. The currents results suggest that residing in rural areas is protective of psychological distress while other studies have observed that people in rural areas present with higher levels of symptoms of psychological distress than their urban counterparts [39, 47]. In South Africa, the high levels of psychological distress may be linked to the persistently high level of HIV prevalence in urban settings [29]. This highlights the importance of integrating mental health care in HIV-related care in areas where most of the population live with HIV.

## Limitations

While SEM remains a powerful tool for exploratory analysis and for the hypothesis-generating process, the analysis may be limited by the difficulty to describe the relationship between HIV and co-occurring mental disorders that may be present prior to HIV diagnosis. The analysis may also be limited by the possibility that other unmeasured variables may have affected the observed relationship between endogenous and exogenous variables and between endogenous variables. In addition, social desirability response bias due to self-report may have influenced some of the results. The cross-sectional nature of the study prevents causal inference limiting our understandings of the exact nature of the relationship between HIV status and psychological distress. Causal pathways could be better clarified with a longitudinal study design.

## Conclusion

HIV status was found to have a direct effect on psychological distress. We therefore conclude that HIV status mediates the relationship between psychological distress and the exogenous factors such as age, sex, race, education and employment. It is important to buffer the impact of these interrelations through effective psychological distress interventions to improve the health and wellbeing of PLHIV in South Africa. These intervention may include provision of social support, self-esteem enhancement, and improving coping skills. The format and content of such interventions should be context specific. Finally, integration of mental health and HIV services is needed.

## Data Availability

The datasets used and/or analysed during the current study are available from the corresponding author on reasonable request.
